# *N*′-(2-Hydr­oxy-4-methoxy­benzyl­idene)-4-methoxy­benzohydrazide

**DOI:** 10.1107/S1600536809032449

**Published:** 2009-08-22

**Authors:** Xinyou Zhang

**Affiliations:** a Department of Chemistry, Baicheng Normal College, Baicheng 137000, People’s Republic of China

## Abstract

In the title compound, C_16_H_16_N_2_O_4_, the dihedral angle between the two benzene rings is 8.7 (2)°. The mol­ecule adopts an *E* configuration about the C=N bond, with an intra­molecular O—H⋯N hydrogen bond involving the hydr­oxy substituent and the hydrazide N atom. In the crystal structure, adjacent mol­ecules are linked through inter­molecular N—H⋯O hydrogen bonds, forming chains propagating in the *b*-axis direction.

## Related literature

For related structures, see: Alhadi *et al.* (2008 ▶); Küçükgüzel *et al.* (2003 ▶); Mohd Lair *et al.* (2009*a* ▶,*b* ▶); Li *et al.* (2009 ▶); Zhang *et al.* (2009 ▶). For a similar hydrazone compound, see: Zhang (2009 ▶). For reference structural data, see: Allen *et al.* (1987 ▶).
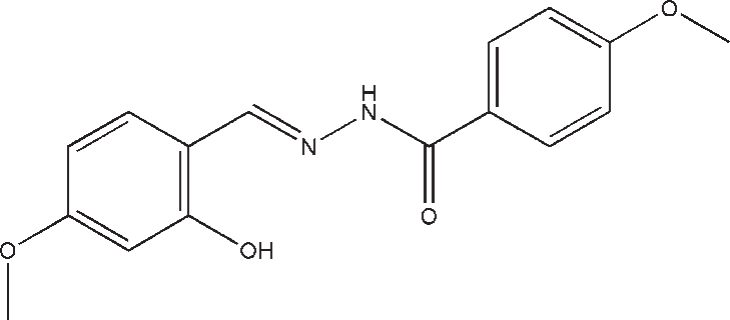


## Experimental

### 

#### Crystal data

C_16_H_16_N_2_O_4_
*M* *_r_* = 300.31
Monoclinic, 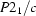

*a* = 17.692 (2) Å
*b* = 5.4131 (7) Å
*c* = 14.933 (2) Å
β = 97.431 (7)°
*V* = 1418.1 (3) Å^3^
*Z* = 4
Mo *K*α radiation
μ = 0.10 mm^−1^
*T* = 298 K
0.23 × 0.20 × 0.20 mm

#### Data collection

Bruker SMART CCD area-detector diffractometer
Absorption correction: multi-scan (*SADABS*; Sheldrick, 1996 ▶) *T*_min_ = 0.977, *T*_max_ = 0.980
8164 measured reflections
3069 independent reflections
2001 reflections with *I* > 2σ(*I*)
*R*_int_ = 0.033

#### Refinement

*R*[*F* ^2^ > 2σ(*F* ^2^)] = 0.046
*wR*(*F* ^2^) = 0.129
*S* = 1.03
3069 reflections
205 parameters
1 restraint
H atoms treated by a mixture of independent and constrained refinement
Δρ_max_ = 0.16 e Å^−3^
Δρ_min_ = −0.16 e Å^−3^

### 

Data collection: *SMART* (Bruker, 2007 ▶); cell refinement: *SAINT* (Bruker, 2007 ▶); data reduction: *SAINT*; program(s) used to solve structure: *SHELXS97* (Sheldrick, 2008 ▶); program(s) used to refine structure: *SHELXL97* (Sheldrick, 2008 ▶); molecular graphics: *SHELXTL* (Sheldrick, 2008 ▶); software used to prepare material for publication: *SHELXL97*.

## Supplementary Material

Crystal structure: contains datablocks global, I. DOI: 10.1107/S1600536809032449/su2137sup1.cif

Structure factors: contains datablocks I. DOI: 10.1107/S1600536809032449/su2137Isup2.hkl

Additional supplementary materials:  crystallographic information; 3D view; checkCIF report

## Figures and Tables

**Table 1:** Hydrogen-bond geometry (Å, °)

*D*—H⋯*A*	*D*—H	H⋯*A*	*D*⋯*A*	*D*—H⋯*A*
O1—H1⋯N1	0.82	1.86	2.577 (2)	146
N2—H2⋯O2^i^	0.90 (1)	2.393 (11)	3.281 (2)	168 (2)

Symmetry code: (i) 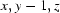.

**Fig. 1.**
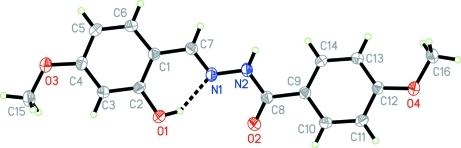
A view of the molecular structure of the title compound, showing 30% displacement ellipsoids. The intramolecular N-H···O hydrogen bond is shown as a dashed line.

**Fig. 2.**
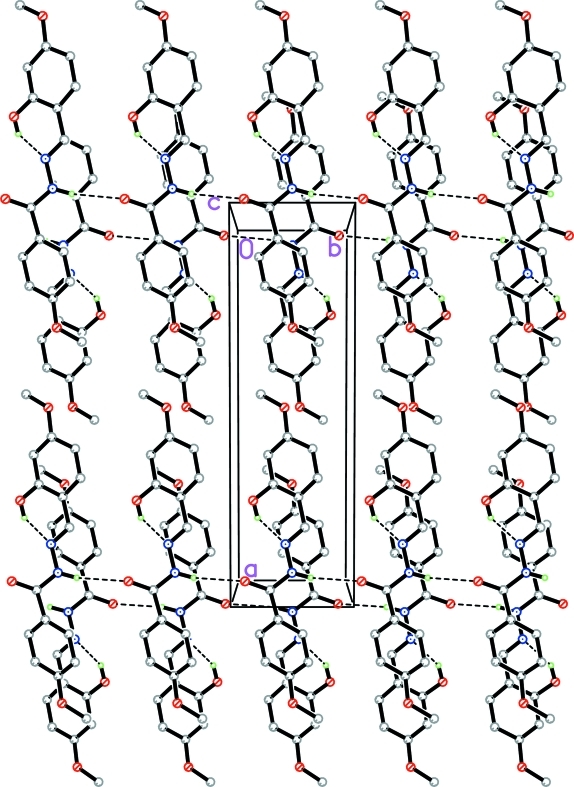
A perspective view, along the c-axis, illustrating the infinite chain structure of the title compound. O-H···N and N-H···O hydrogen bonds are shown as dashed lines [see Table 1 for details; H atoms not involved in hydrogen bonding have been omitted for clarity].

## supplementary crystallographic information

### Comment

Hydrazone compounds are readily synthesized by the reaction of aldehydes with hydrazides. A large number of hydrazone compounds have been reported (Alhadi *et al.*, 2008; Küçükgüzel *et al.*, 2003; Li *et al.*, 2009; Zhang *et al.*, 2009). Recently, the author reported on the crystal structure of a hydrazone compound derived from the reaction of 2-methoxybenzaldehyde with 4-methoxybenzohydrazide (Zhang, 2009). Herein, the crystal structure of the new title hydrazone, prepared from the reaction of 2-hydroxy-4-methoxybenzaldehyde with 4-methoxybenzohydrazide, is reported on.

The molecule structure of the title compound is illustrated in Fig. 1. The molecule adopts an *E* configuration about the C═N bond. The dihedral angle involving the two benzene rings is 8.7 (2)°. There is an intramolecular O1—H1···N1 hydrogen bond, involving the hydroxyl substituent and the hydrazide N-atom (Table 1). All the bond lengths are within normal values (Allen *et al.*, 1987) and are comparable with those observed in similar compounds (Mohd Lair *et al.*, 2009*a*,b; Zhang, 2009).

In the crystal structure of the title compound adjacent molecules are linked through intermolecular N—H···O hydrogen bonds, forming chains propagating in b direction (Table 1 and Fig. 2).

### Experimental

2-Hydroxy-4-methoxybenzaldehyde (1.0 mmol, 152.2 mg) and 4-methoxybenzohydrazide (1.0 mmol, 166.2 mg) were mixed in a methanol solution, and the mixture was refluxed for 1 h. Colorless block-shaped crystals of the title compound were formed by slow evaporation of the solution in air.

### Refinement

Atom H2 attached to N2 was located from a difference electron-density map and freely refined with *U*_iso_(H) restrained to 0.08 (2) Å^2^. The other H-atoms were included in calculated positions and refined as riding atoms: d(C—H) = 0.93–0.96 Å, d(O—H) = 0.82 Å, with *U*_iso_(H) = 1.2*U*_eq_(C) and 1.5*U*_eq_(C_methyl_ and O).

### Crystal data

**Table:**

C_16_H_16_N_2_O_4_	*F*(000) = 632
*M**_r_* = 300.31	*D*_x_ = 1.407 Mg m^−^^3^
Monoclinic, *P*2_1_/*c*	Mo *K*α radiation, λ = 0.71073 Å
Hall symbol: -P 2ybc	Cell parameters from 1647 reflections
*a* = 17.692 (2) Å	θ = 2.3–24.5°
*b* = 5.4131 (7) Å	µ = 0.10 mm^−^^1^
*c* = 14.933 (2) Å	*T* = 298 K
β = 97.431 (7)°	Block, colorless
*V* = 1418.1 (3) Å^3^	0.23 × 0.20 × 0.20 mm
*Z* = 4	

### Data collection

**Table:**

Bruker SMART CCD area-detector diffractometer	3069 independent reflections
Radiation source: fine-focus sealed tube	2001 reflections with *I* > 2σ(*I*)
graphite	*R*_int_ = 0.033
ω scans	θ_max_ = 27.0°, θ_min_ = 2.3°
Absorption correction: multi-scan (*SADABS*; Sheldrick, 1996)	*h* = −21→22
*T*_min_ = 0.977, *T*_max_ = 0.980	*k* = −6→6
8164 measured reflections	*l* = −17→19

### Refinement

**Table:**

Refinement on *F*^2^	Primary atom site location: structure-invariant direct methods
Least-squares matrix: full	Secondary atom site location: difference Fourier map
*R*[*F*^2^ > 2σ(*F*^2^)] = 0.046	Hydrogen site location: inferred from neighbouring sites
*wR*(*F*^2^) = 0.129	H atoms treated by a mixture of independent and constrained refinement
*S* = 1.03	*w* = 1/[σ^2^(*F*_o_^2^) + (0.0559*P*)^2^ + 0.2712*P*] where *P* = (*F*_o_^2^ + 2*F*_c_^2^)/3
3069 reflections	(Δ/σ)_max_ < 0.001
205 parameters	Δρ_max_ = 0.16 e Å^−^^3^
1 restraint	Δρ_min_ = −0.16 e Å^−^^3^

### Special details

**Table:**

Geometry. All e.s.d.'s (except the e.s.d. in the dihedral angle between two l.s. planes) are estimated using the full covariance matrix. The cell e.s.d.'s are taken into account individually in the estimation of e.s.d.'s in distances, angles and torsion angles; correlations between e.s.d.'s in cell parameters are only used when they are defined by crystal symmetry. An approximate (isotropic) treatment of cell e.s.d.'s is used for estimating e.s.d.'s involving l.s. planes.
Refinement. Refinement of *F*^2^ against ALL reflections. The weighted *R*-factor *wR* and goodness of fit *S* are based on *F*^2^, conventional *R*-factors *R* are based on *F*, with *F* set to zero for negative *F*^2^. The threshold expression of *F*^2^ > σ(*F*^2^) is used only for calculating *R*-factors(gt) *etc*. and is not relevant to the choice of reflections for refinement. *R*-factors based on *F*^2^ are statistically about twice as large as those based on *F*, and *R*- factors based on ALL data will be even larger.

### Fractional atomic coordinates and isotropic or equivalent isotropic displacement parameters (Å^2^)

**Table:**

	*x*	*y*	*z*	*U*_iso_*/*U*_eq_	
O1	0.25296 (7)	0.8356 (2)	0.42022 (10)	0.0497 (4)	
H1	0.2079	0.7986	0.4079	0.075*	
O2	0.03919 (7)	0.9056 (3)	0.38193 (10)	0.0550 (4)	
O3	0.49908 (7)	0.5665 (3)	0.36425 (10)	0.0584 (4)	
O4	−0.29179 (7)	0.4958 (3)	0.40281 (9)	0.0479 (4)	
N1	0.13948 (8)	0.5576 (3)	0.35985 (10)	0.0426 (4)	
N2	0.06360 (9)	0.5020 (3)	0.35871 (12)	0.0449 (4)	
C1	0.26537 (10)	0.4485 (3)	0.34327 (12)	0.0375 (4)	
C2	0.29666 (10)	0.6628 (3)	0.38635 (12)	0.0381 (4)	
C3	0.37456 (10)	0.7056 (3)	0.39548 (13)	0.0419 (5)	
H3	0.3949	0.8454	0.4258	0.050*	
C4	0.42197 (10)	0.5403 (4)	0.35937 (13)	0.0425 (5)	
C5	0.39204 (11)	0.3296 (4)	0.31433 (13)	0.0464 (5)	
H5	0.4237	0.2202	0.2889	0.056*	
C6	0.31544 (11)	0.2860 (4)	0.30805 (13)	0.0437 (5)	
H6	0.2959	0.1430	0.2793	0.052*	
C7	0.18490 (10)	0.3929 (4)	0.33728 (12)	0.0422 (5)	
H7	0.1665	0.2389	0.3172	0.051*	
C8	0.01619 (10)	0.6911 (4)	0.37443 (12)	0.0407 (4)	
C9	−0.06413 (10)	0.6228 (3)	0.38074 (12)	0.0375 (4)	
C10	−0.10700 (10)	0.7816 (4)	0.42781 (13)	0.0429 (5)	
H10	−0.0843	0.9226	0.4548	0.052*	
C11	−0.18213 (11)	0.7328 (4)	0.43479 (13)	0.0435 (5)	
H11	−0.2100	0.8399	0.4666	0.052*	
C12	−0.21659 (10)	0.5231 (3)	0.39429 (12)	0.0374 (4)	
C13	−0.17495 (10)	0.3632 (4)	0.34784 (13)	0.0437 (5)	
H13	−0.1979	0.2228	0.3206	0.052*	
C14	−0.09899 (10)	0.4126 (4)	0.34193 (13)	0.0436 (5)	
H14	−0.0709	0.3030	0.3114	0.052*	
C15	0.53412 (11)	0.7685 (4)	0.41459 (18)	0.0660 (7)	
H15A	0.5154	0.9209	0.3873	0.099*	
H15B	0.5884	0.7599	0.4150	0.099*	
H15C	0.5222	0.7609	0.4754	0.099*	
C16	−0.32941 (11)	0.2764 (4)	0.36696 (15)	0.0531 (5)	
H16A	−0.3256	0.2651	0.3035	0.080*	
H16B	−0.3821	0.2823	0.3759	0.080*	
H16C	−0.3057	0.1346	0.3974	0.080*	
H2	0.0500 (13)	0.341 (2)	0.3594 (16)	0.080*	

### Atomic displacement parameters (Å^2^)

**Table:**

	*U*^11^	*U*^22^	*U*^33^	*U*^12^	*U*^13^	*U*^23^
O1	0.0404 (8)	0.0411 (8)	0.0694 (9)	0.0031 (6)	0.0135 (7)	−0.0102 (7)
O2	0.0463 (8)	0.0446 (9)	0.0753 (10)	−0.0066 (7)	0.0117 (7)	−0.0086 (8)
O3	0.0352 (8)	0.0575 (10)	0.0830 (11)	0.0003 (6)	0.0101 (7)	−0.0097 (8)
O4	0.0360 (7)	0.0486 (8)	0.0610 (9)	0.0001 (6)	0.0130 (6)	−0.0039 (7)
N1	0.0330 (8)	0.0451 (10)	0.0502 (9)	0.0006 (7)	0.0069 (7)	0.0031 (8)
N2	0.0322 (8)	0.0430 (10)	0.0598 (10)	−0.0019 (7)	0.0074 (7)	0.0010 (9)
C1	0.0369 (10)	0.0346 (10)	0.0410 (10)	0.0016 (8)	0.0054 (8)	0.0025 (8)
C2	0.0395 (10)	0.0329 (10)	0.0429 (10)	0.0051 (8)	0.0088 (8)	0.0012 (8)
C3	0.0398 (10)	0.0364 (11)	0.0494 (11)	−0.0002 (8)	0.0048 (8)	−0.0029 (9)
C4	0.0333 (10)	0.0451 (12)	0.0496 (11)	0.0048 (8)	0.0069 (8)	0.0050 (9)
C5	0.0439 (11)	0.0431 (12)	0.0531 (12)	0.0092 (9)	0.0095 (9)	−0.0035 (10)
C6	0.0442 (11)	0.0358 (11)	0.0508 (11)	0.0026 (8)	0.0052 (9)	−0.0037 (9)
C7	0.0409 (11)	0.0375 (11)	0.0476 (11)	−0.0024 (8)	0.0030 (8)	−0.0010 (9)
C8	0.0372 (10)	0.0443 (12)	0.0407 (10)	−0.0016 (9)	0.0051 (8)	−0.0024 (9)
C9	0.0349 (9)	0.0374 (10)	0.0401 (10)	0.0016 (8)	0.0043 (8)	0.0009 (8)
C10	0.0426 (11)	0.0377 (11)	0.0482 (11)	0.0003 (8)	0.0046 (9)	−0.0071 (9)
C11	0.0453 (11)	0.0403 (11)	0.0459 (11)	0.0075 (9)	0.0093 (9)	−0.0057 (9)
C12	0.0341 (9)	0.0388 (11)	0.0398 (10)	0.0034 (8)	0.0061 (7)	0.0028 (8)
C13	0.0399 (11)	0.0380 (11)	0.0534 (12)	−0.0016 (8)	0.0064 (9)	−0.0093 (9)
C14	0.0390 (10)	0.0405 (11)	0.0521 (11)	0.0032 (8)	0.0096 (9)	−0.0096 (9)
C15	0.0375 (12)	0.0611 (15)	0.0982 (19)	−0.0027 (10)	0.0041 (11)	−0.0059 (14)
C16	0.0390 (11)	0.0527 (13)	0.0678 (14)	−0.0066 (9)	0.0072 (10)	−0.0012 (11)

### Geometric parameters (Å, °)

**Table:**

O1—C2	1.353 (2)	C6—H6	0.9300
O1—H1	0.8200	C7—H7	0.9300
O2—C8	1.231 (2)	C8—C9	1.483 (2)
O3—C4	1.364 (2)	C9—C14	1.385 (3)
O3—C15	1.423 (3)	C9—C10	1.395 (2)
O4—C12	1.361 (2)	C10—C11	1.372 (2)
O4—C16	1.431 (2)	C10—H10	0.9300
N1—C7	1.274 (2)	C11—C12	1.389 (3)
N1—N2	1.374 (2)	C11—H11	0.9300
N2—C8	1.363 (2)	C12—C13	1.380 (2)
N2—H2	0.903 (10)	C13—C14	1.384 (2)
C1—C6	1.398 (2)	C13—H13	0.9300
C1—C2	1.405 (3)	C14—H14	0.9300
C1—C7	1.447 (2)	C15—H15A	0.9600
C2—C3	1.387 (2)	C15—H15B	0.9600
C3—C4	1.383 (2)	C15—H15C	0.9600
C3—H3	0.9300	C16—H16A	0.9600
C4—C5	1.393 (3)	C16—H16B	0.9600
C5—C6	1.367 (3)	C16—H16C	0.9600
C5—H5	0.9300		
			
C2—O1—H1	109.5	C14—C9—C10	118.38 (17)
C4—O3—C15	118.20 (15)	C14—C9—C8	123.83 (17)
C12—O4—C16	117.91 (14)	C10—C9—C8	117.79 (17)
C7—N1—N2	119.46 (17)	C11—C10—C9	120.98 (18)
C8—N2—N1	117.10 (16)	C11—C10—H10	119.5
C8—N2—H2	123.5 (15)	C9—C10—H10	119.5
N1—N2—H2	118.2 (15)	C10—C11—C12	119.92 (17)
C6—C1—C2	117.41 (16)	C10—C11—H11	120.0
C6—C1—C7	120.80 (17)	C12—C11—H11	120.0
C2—C1—C7	121.77 (16)	O4—C12—C13	124.80 (17)
O1—C2—C3	117.15 (17)	O4—C12—C11	115.29 (16)
O1—C2—C1	122.13 (16)	C13—C12—C11	119.90 (17)
C3—C2—C1	120.71 (16)	C12—C13—C14	119.78 (18)
C4—C3—C2	119.92 (18)	C12—C13—H13	120.1
C4—C3—H3	120.0	C14—C13—H13	120.1
C2—C3—H3	120.0	C13—C14—C9	121.03 (17)
O3—C4—C3	124.39 (18)	C13—C14—H14	119.5
O3—C4—C5	115.21 (16)	C9—C14—H14	119.5
C3—C4—C5	120.40 (17)	O3—C15—H15A	109.5
C6—C5—C4	119.08 (17)	O3—C15—H15B	109.5
C6—C5—H5	120.5	H15A—C15—H15B	109.5
C4—C5—H5	120.5	O3—C15—H15C	109.5
C5—C6—C1	122.43 (18)	H15A—C15—H15C	109.5
C5—C6—H6	118.8	H15B—C15—H15C	109.5
C1—C6—H6	118.8	O4—C16—H16A	109.5
N1—C7—C1	119.14 (18)	O4—C16—H16B	109.5
N1—C7—H7	120.4	H16A—C16—H16B	109.5
C1—C7—H7	120.4	O4—C16—H16C	109.5
O2—C8—N2	121.38 (17)	H16A—C16—H16C	109.5
O2—C8—C9	122.52 (17)	H16B—C16—H16C	109.5
N2—C8—C9	116.10 (17)		

### Hydrogen-bond geometry (Å, °)

**Table:**

*D*—H···*A*	*D*—H	H···*A*	*D*···*A*	*D*—H···*A*
O1—H1···N1	0.82	1.86	2.577 (2)	146
N2—H2···O2^i^	0.90 (1)	2.39 (1)	3.281 (2)	168 (2)

Symmetry codes: (i) *x*, *y*−1, *z*.
